# Increased Seizure Susceptibility in Mice 30 Days after Fluid Percussion Injury

**DOI:** 10.3389/fneur.2013.00028

**Published:** 2013-03-21

**Authors:** Sanjib Mukherjee, Suzanne Zeitouni, Clarissa Fantin Cavarsan, Lee A. Shapiro

**Affiliations:** ^1^Department of Surgery, Scott and White HospitalTemple, TX, USA; ^2^Central Texas Veterans Health Care SystemTemple, TX, USA; ^3^Department of Surgery, College of Medicine, Texas A&M Health Science CenterTemple, TX, USA; ^4^Department of Neuroscience and Experimental Therapeutics, College of Medicine, Texas A&M Health Science CenterTemple, TX, USA

**Keywords:** traumatic brain injury, post-traumatic epilepsy, pentylenetetrazole, mouse models

## Abstract

Traumatic brain injury (TBI) has been reported to increase seizure susceptibility and also contribute to the development of epilepsy. However, the mechanistic basis of the development of increased seizure susceptibility and epilepsy is not clear. Though there is substantial work done using rats, data are lacking regarding the use of mice in the fluid percussion injury (FPI) model. It is unclear if mice, like rats, will experience increased seizure susceptibility following FPI. The availability of a mouse model of increased seizure susceptibility after FPI would provide a basis for the use of genetically modified mice to study mechanism(s) of the development of post-traumatic epilepsy. Therefore, this study was designed to test the hypothesis that, mice subjected to a FPI develop increased seizure susceptibility to a subconvulsive dose of the chemoconvulsant, pentylenetetrazole (PTZ). Three groups of mice were used: FPI, sham, and naïve controls. On day 30 after FPI, mice from the three groups were injected with PTZ. The results showed that FPI mice exhibited an increased severity, frequency, and duration of seizures in response to PTZ injection compared with the sham and naïve control groups. Histopathological assessment was used to characterize the injury at 1, 3, 7, and 30 days after FPI. The results show that mice subjected to the FPI had a pronounced lesion and glial response that was centered at the FPI focus and peaked at 3 days. By 30 days, only minimal evidence of a lesion is observed, although there is evidence of a chronic glial response. These data are the first to demonstrate an early increase in seizure susceptibility following FPI in mice. Therefore, future studies can incorporate transgenic mice into this model to further elucidate mechanisms of TBI-induced increases in seizure susceptibility.

## Introduction

An estimated 1.7 million people in the U.S. experience a traumatic brain injury (TBI) each year, 80,000 of which develop long-term disabilities and 50,000 of which are fatal (Faul, 2010). Approximately 3.2 million individuals are living with such disabilities in the U.S., resulting in a large economic burden, primarily through loss of work and medical expenses (Finkelstein et al., 2006). TBI causes several neuropathological manifestations, including cognitive, emotional, physiological, and psychological deficits (Rosenthal et al., 1998; Junqué, 1999; Vakil, 2005; Nampiaparampil, 2008; Bales et al., 2009). In addition, to these deficits, another pathology often associated with TBI is increased seizure susceptibility and the development of epilepsy (D’Ambrosio and Perucca, 2004). TBI is responsible for the development of 10–20% of symptomatic epilepsy in the general population (Pitkänen and Bolkvadze, 2012) and has also been reported to increase seizure susceptibility (Kharatishvili and Pitkänen, 2010). Why TBI results in the development of epilepsy and increased seizure susceptibility remains largely unknown, although several candidate mechanisms have been postulated, including: neurodegeneration, neuroplasticity, neuroinflammation, and connective tissue formation. The use of animal models that mimic these effects will aid in the understanding of the mechanisms of TBI and may provide help in the development of better treatment strategies.

Previous studies in rats using the fluid percussion injury (FPI) model have demonstrated an increase in seizure susceptibility, as measured by a second-hit chemoconvulsant challenge, as well as the development of spontaneous epileptiform discharges, the hallmark of epilepsy (Silva et al., 2011; Kharatishvili et al., 2006). One of the main benefits of this model in rats is the high reproducibility. However, because this model has not been extended to mice, it lacks the benefits of using different genetic models. Fundamental studies are needed to enable mechanistic studies using transgenic mice in the FPI model. Therefore, this study examined second-hit seizure susceptibility in mice, using Pentylenetetrazol (PTZ) at 30 days after FPI. To further characterize this mouse model of FPI, histopathological and glial response data are also provided.

## Materials and Methods

### Strain and surgeries

All experimental protocols were carried out as previously approved by the Institutional Animal Care Committee (IACUC) of Texas A&M University Health Science Center and Scott &White hospital. Male C57Bl6 mice from Charles River were used in these studies. All the mice from FPI and sham groups underwent surgery. Mice were initially anesthetized with 4% isoflurane and oxygen for anesthesia induction and later to 2% isoflurane for maintenance. Once under anesthesia, the heads of the animals were shaved. Strict sterile technique was maintained during surgical procedures. Animals were placed in a stereotaxic instrument with an attachment for mouse surgery (Stoelting, Inc., IL, USA). A 2-mm hole was drilled, with dura intact, in the skull over the left parietal cortex (antero-posterior: +1.5 mm; medio-lateral: −1.2 mm). A female luer-lock (PlasticOne) was connected to the hole in the skull. Animals in the FPI group received a pressure pulse of 1.5–1.7 atm from the FPI apparatus through the luer-lock for 12–16 ms. Sham animals received identical treatment except no pressure pulse was delivered. Naïve animals were not surgically manipulated. Animals were housed singly after FPI with a 12-h light–dark cycle (light on 6:00 and light off 18:00). All animals had continuous access to food and water.

### Histopathology

Forty C57Bl6 male mice were used for histological examination. Animals were randomly assigned to experimental (*N* = 16), sham (*N* = 16), and naïve control (*N* = 8) groups. In order to define the injury and subsequent inflammatory response, separate groups of mice were sacrificed at 1, 3, 7, and 30 days after FPI (*N* = 4 sham, 4 FPI, and 2 Naïve mice per time point). Mice were euthanized via a transcardiac perfusion of saline followed by paraformaldehyde (PFA) as previously described (Arisi et al., 2011). Briefly, animals were given an overdose of i.p. Euthasol, followed by an incision in the right atrium while simultaneously pumping 0.9% sterile saline through the left ventricle. After the blood ran clear (∼50 ml), 4.0% PFA was pumped through the left ventricle. Brains were allowed to post-fix in the skull for 24 h following perfusion, after which they were removed, and post-fixed for another 24 h in PFA. Brains were subsequently hemi-sectioned and cut at 50 μm for analysis. Gross examination of the impact lesion was performed upon extraction from the skull, prior to cutting, in addition to histological and immunocytochemical analysis.

#### Cresyl violet

Sections were mounted onto gelatin-coated slides and allowed to dry overnight. Slides were then dehydrated and defatted in 70, 95, and 100% ETOH, followed by rehydration and staining in the cresyl violet solution (Sigma, St Louis, MO, USA). Slides were rinsed in de-ionized H_2_O, again dehydrated, cleared with xylenes, and coverslips were applied using permount. Sections were then visualized using a Leica SCN 400 (Leica Corp., Wetzlar, Germany) slide scanner.

#### Fluoro-Jade C histology for damaged cells

Sections were mounted onto gelatin-coated slides and Fluoro-Jade C staining took place according to the packaging instructions (AG325, Millipore Inc., Billerica, MA, USA). Once these slides were dry, they were immersed in xylenes and then cover slips were applied using DPX mounting media. Sections were then visualized on a Olympus IX81 (Olympus Inc., Center Valley, PA, USA) inverted microscope equipped to visualize FITC.

#### GFAP and Iba1 immunocytochemistry for astrocytes and microglia

Sections were reacted free-floating as previously described (Shapiro et al., 2008, 2009). Briefly, fluorescent labeling of both antibodies was performed in order to provide a qualitative temporal description of the inflammatory response in the ipsi and contralateral cortex following FPI. For GFAP-labeling, a fluorescent-tagged primary GFAP antibody (1:2000; Sigma #C9205) was used for analysis. For Iba1, a rabbit polyclonal antibody (1:500; Wako labs # 019-19741) was used, followed by fluorescent-conjugated goat anti-rabbit IgG (Alexa-fluor 555; Invitrogen Inc.). Sections were then visualized on a Olympus IX81 (Olympus Inc.) inverted, laser-scanning confocal microscope. In addition, we performed a peroxidase reaction using DAB for GFAP (Rabbit polyclonal 1:1000; Sigma#G9269) and these slides were visualized on the Leica SCN 400 slide scanner (Leica Corp.).

### PTZ second-hit seizure challenge

Twenty three male C57Bl/6 mice (23–28 g) were used in this part of the study. The 30-day post-FPI timepoint was selected because previous studies using other models of epileptogenesis have examined the 30-day timepoint for increased seizure susceptibility (Blanco et al., 2009; Wilhelm et al., 2012). Animals were randomly assigned to experimental (*N* = 9), sham (*N* = 9), and naïve control (*N* = 5) groups. To test for seizure susceptibility, 30 days after the surgery, all the animals from FPI, sham, and naïve control groups were injected i.p. with a subconvulsive dose (Jain et al., 2011) of PTZ (30 mg/kg; Sigma). Immediately following the single injection of PTZ, mice were monitored and videotaped, and seizure scores were calculated for 20 min by reviewers blind to the condition of the animal. Seizures were scored as per a modified Racine Scale (Shapiro et al., 2005). Briefly, stage 1 seizures were classified by movement of mouth and facial muscles; stage 2 seizures were classified as head-bobs and rocking; stage 3 seizures were classified by forelimb clonus; stage 4 seizures were classified as forelimb and hindlimb clonus; stage 5 seizures were classified as tonic clonic activity and loss of balance. The seizure parameters that were examined in this study were: severity, frequency, and duration of seizures. Data was analyzed by contingency table analysis with Chi-Square, using SPSS 9.0.

## Results

### Gross examination

Gross examination of the brain following removal revealed a substantial lesion surrounded by blood in mice that received the FPI. The lesion was present both at the 1- and 3-day timepoints (data not shown). This was not observed in the sham animals. The blood surrounding the lesion site appeared to be more prevalent at 3 days relative to the 1-day timepoint. By 7 days, the lesion was still present, but the blood surrounding the lesion was no longer evident. At 30 days, there was no gross evidence of a lesion.

### Histopathology

**Figure 1:**
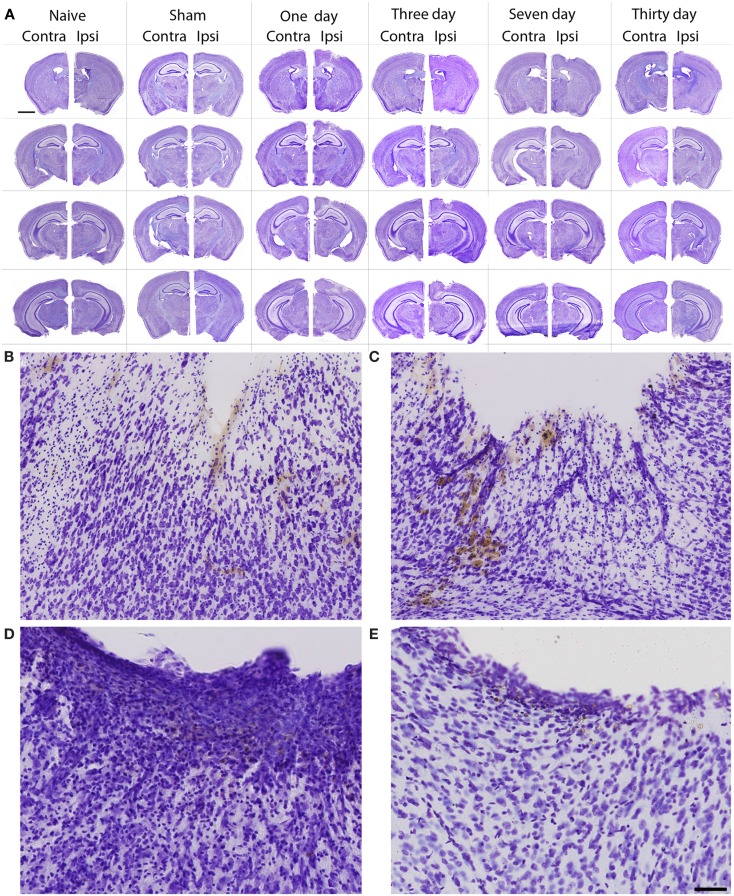
**Cresyl violet stained tissue from FPI mice, sham mice, and naïve mice**. In **(A)**, low magnification images are shown to illustrate the location and span of the lesion resulting from the FPI. Note that the sham column depicts each of the timepoints (1–30 days in descending order) in the central hippocampus. Note that the dural surface is intact in the sham animals. In **(B–E)**, high magnification images are shown from the FPI mice. In **(B)**, the lesion area is enlarged from the 1-day post-FPI timepoint. Tissue damage is clearly evident. The brown reaction product is likely a reaction to the iron associated with hemoglobin of erythrocytes. Since blood degradation products are ingested by macrophages, the cells containing the brown staining are quite possibly macrophages. Note that the cortical layers subjacent to the lesion appear relatively intact at this timepoint. In **(C)**, the boxed area from the 3-day FPI mice is shown in enlargement. In **(D)**, the dark blue staining is likely indicative of pial repair. Note that the typical layering and columnar appearance of the cortex is altered. At 30 days post-FPI **(E)**, the pial surface appears intact, but remnants of blood in the peri-lesion area remain. Note that the typical layering of the cortex remains altered at this timepoint. Scale bar in **(A)** = 2 mm for all low magnification images and scale bar in in **(E)** = 80 μm for **(B–E)**.

**Figure 2:**
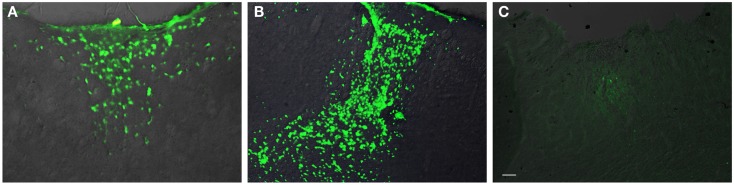
**Fluoro-Jade labeling in peri-lesion area following FPI**. In **(A)**, degenerating cells are observed in the immediate peri-lesion region at 1-day post-FPI. In **(B)**, degeneration is considerably more robust at the 3-day post-FPI compared to the 1-day post-FPI timepoint. By 7 days post-FPI **(C)**, the number of degenerating cells is considerably less (and was non-existent in most of the sections examined) than the 1- or 3-day post-FPI timepoints. Scale bar in **(C)** = 50 μm for all images.

In general, the FPI results in a consistent lesion that is focused around the center of the impact area and emanates deep to, lateral and medial to the impact area. Examination of Cresyl Violet stained tissue sections revealed that at 1 day after FPI, the lesion size spanned from Anterior/Posterior (AP) +0.3 to −2.54 mm from bregma. The average medial to lateral (ML) span was 1.82 mm (±0.42 mm) at its widest margin, which was located around the epicenter of the impact (AP −1.2 mm, ML +1.5 mm from bregma). The median ML length was 1.78 mm. The range of the dorsal/ventral (DV) damage was between 0.18 and 1.09 mm from the pial surface (Figure 1). Leukocyte infiltration was observed in the region surrounding the lesion (Figure 1). Fluoro-Jade staining was performed and damaged cells were seen immediately lateral, medial, and subjacent to the impact zone (Figure 2) at the 1 day post-FPI timepoint.

**Figure 3:**
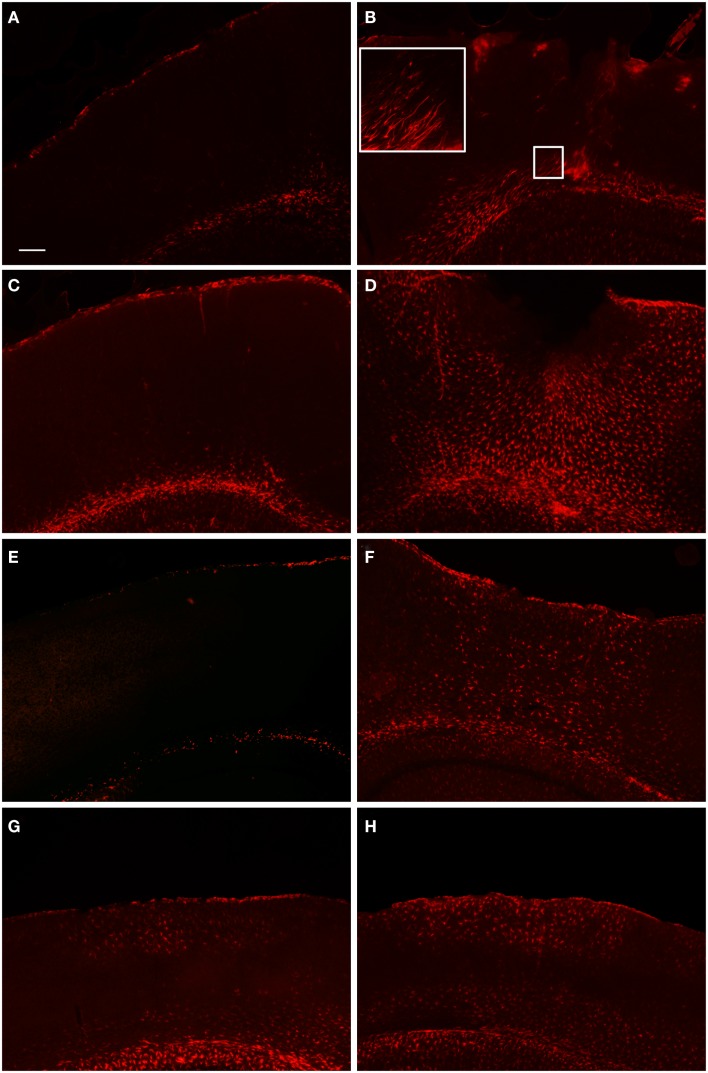
**Fluorescent microscopy of GFAP-labeling in the peri-lesion area and corresponding contralateral hemisphere**. At 1 day after FPI **(A,B)**, there is only a minimal astrocyte reaction in the peri-lesion area in the ipsilateral cortex **(B)**. Note in the inset image, the elongated appearance of these astrocytes oriented toward the lesion emanating from the area of the corpus callosum, or possibly the underlying lateral ventricle. This morphology, coupled with the minimal astrocytic staining in the peri-lesion area, is indicative of the early stages of astrocyte activation. In the corresponding contralateral hemisphere, minimal GFAP-labeling is observed. At 3 days post-FPI **(C,D)**, a robust number of GFAP-labeled cells with an activated appearance are observed in the peri-lesion area **(D)**. Only minimal GFAP-labeling is observed in the contralateral hemisphere at this timepoint **(C)**. At 7 days post-FPI **(E,F)**, the appearance of GFAP-labeled astrocytes with an activated appearance is decreased relative to the 3-day timepoint. In the corresponding contralateral hemisphere at 7 days post-FPI, there appears to be diminished GFAP-labeling relative to 1 and 3 days, as well as sham and naïve mice (data not shown). At 30 days post-FPI **(G,H)**, a sizable population of GFAP-labeled astrocytes is observed in both the contralateral **(G)** and ipsilateral **(H)** hemispheres. In both hemispheres, the labeling is quite robust in layers I–IV and VI, but conspicuously absent in layer V. Scale bar in **(A)** = 50 μm for all images.

At 3 days post-FPI, the lesion spanned from AP +0.26 to −2.60 mm from bregma. The average ML span was 1.44 mm (±0.47 mm) at its widest margin, which was located around the epicenter of the impact (AP −1.2 mm, ML +1.5 mm from bregma). The median ML length was 1.31 mm (Figure 3). The range of the DV damage was between 0.15 and 0.86 mm from the pial surface. Leukocyte infiltration was observed in the region surrounding the lesion (Figure 1). Fluoro-Jade staining was performed and damaged cells were seen extending deep into the tissue, reaching as far ventral as the corpus callosum (Figure 2) at the 3-day post-FPI timepoint.

At 7 days post-FPI, the lesion size was noticeably smaller relative to the 1- and 3-day timepoints. In two of the four FPI animals examined, the pial surface had healed such that no breach was evident. In the other two mice, only a small disruption of the pial surface was evident. At this timepoint, the lesion size spanned from AP +0.26 to −2.54 mm from bregma. The average ML span was 1.08 mm (±0.28 mm) at its widest margin, which was located around the epicenter of the impact (AP −1.2 mm, ML +1.5 mm from bregma). The median ML length was 1.28 mm (Figure 1). The range of the DV damage was between 0.43 and 0.65 mm from the pial surface. Fluoro-Jade staining detected only a small number of Fluoro-Jade cells at the 7-day post-FPI timepoint. These cells were located in the immediate surrounding area of the impact zone (Figure 2).

At 30 days after FPI, the pial surface appeared to be fully restored and intact in all of the mice examined. Although only minimal superficial evidence of the lesion was evident, the cortical layers within and surrounding the region where the lesion occurred no longer exhibit a clear I–VI pattern (Figure 1). No Fluoro-Jade labeling was found at this timepoint.

### Glial response to FPI

#### GFAP

**Figure 4:**
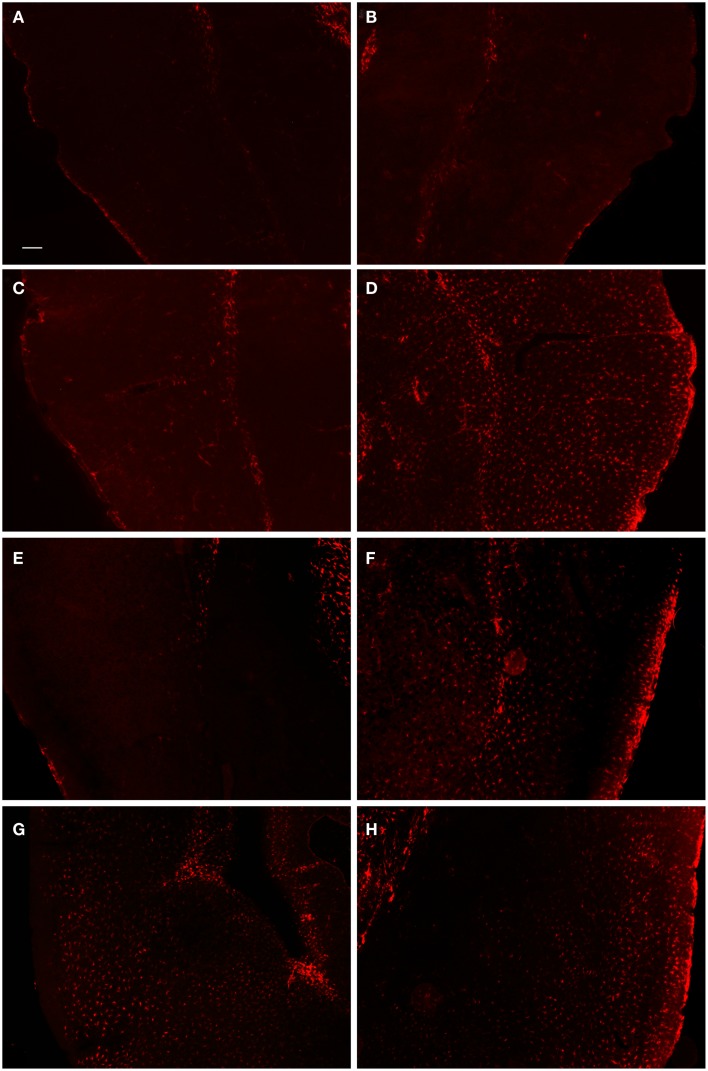
**Fluorescent microscopy of GFAP-labeling in the ipsilateral and contralateral hemispheres**. At 1-day post-FPI, only minimal GFAP-labeling is observed in the contralateral **(A)** and ipsilateral **(B)** hemispheres. At 3 days post-FPI, only minimal GFAP-labeling is observed in the contralateral hemisphere **(C)**, but a robust number of GFAP-labeled astrocytes are observed in the ipsilateral hemisphere **(D)**. This pattern of labeling is also evident at 7 days post-FPI **(E,F)**. It is pertinent to note that in the contralateral hemisphere **(E)**, an overall depletion of GFAP-labeling is observed. This is similar to the observation in Figure 3 **(E,F)**, in which the contralateral hemisphere corresponding to the lesion site also appeared depleted of GFAP-labeling. At 30 days post-FPI **(G,H)**, GFAP-labeled astrocytes are widely distributed throughout both, ipsilateral **(G)** and contralateral **(H)** hemispheres. Note, that we have also provided light microscopic images of DAB-reacted tissue (Figure 5) demonstrating these same observations. Scale bar in **(A)** = 50 μm for all images.

**Figure 5:**
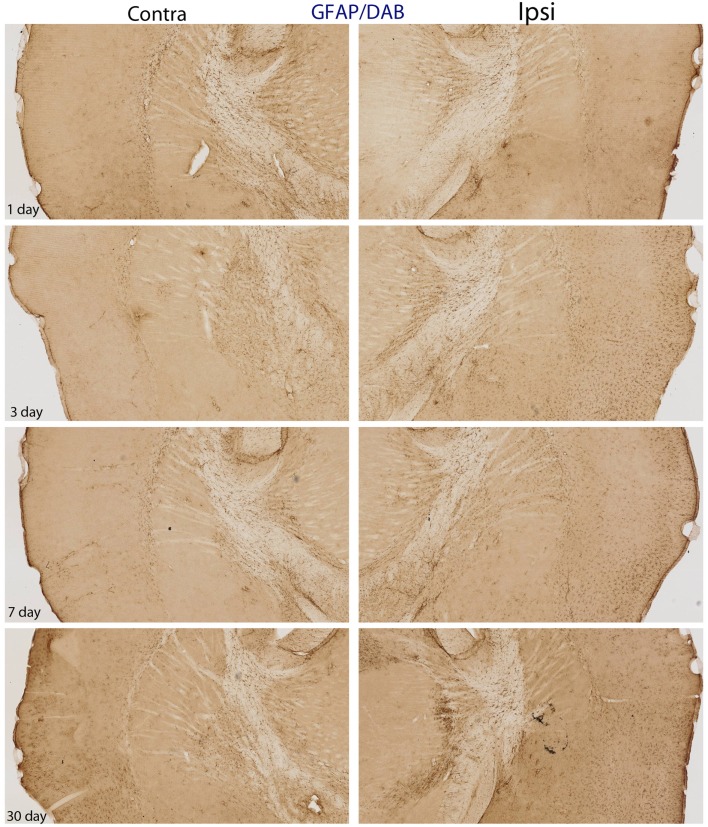
**DAB-reacted GFAP immunohistochemistry**. This figure depicts the same pattern of GFAP staining as that shown in Figure 4. The advantage of the DAB-reacted tissue is that it allows for the reader to appreciate the coordinates of the tissue, as well as the relative staining in the ipsi and contra lateral hemispheres at lower magnification.

**Figure 6:**
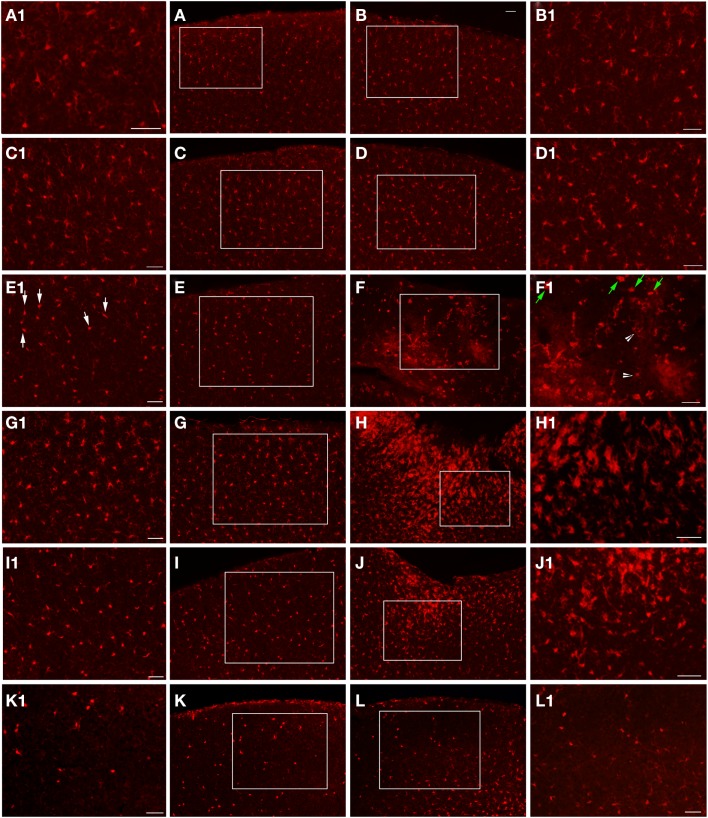
**Epi-fluorescent microscopy of Iba1-labeling following FPI**. In **(A,B)**, images are from a naïve control mouse. In **(C,D)**, images are from a sham mouse. Note the typical distribution of Iba1-labeled microglial cells in both of these animals. Enlargements **(A1–D1)** are provided in order to illustrate the normal appearance of resting microglial cells in the region corresponding to the FPI-induced lesion **(B,D)** and the region corresponding to this area in the contralateral hemisphere **(A,C)**. Note that microglial cells in normal conditions show minimal overlap, with each cell occupying a specific domain within the parenchyma. At 1-day post-FPI **(E,F)**, some of the Iba1-labeled microglial cells in the contralateral hemisphere **(E1)** exhibit the early stages of microglial activation (white arrows). In the peri-lesion region **(F1)**, robust Iba1-labeling is apparent. Many of these labeled cells at this timepoint exhibit a relatively simple morphology (green arrows) and to a lesser extent more complex microglial cells (white arrowheads) are also evident (Shapiro et al., 2008). At 3 days post-FPI **(G,H)**, microglial activation is increased in the contralateral hemisphere **(G)** relative to 1-day post-FPI. In the peri-lesion region **(H)**, a robust Iba1-labeling is observed. At 7 days post-FPI **(I,J)**, the distribution and appearance of Iba1-labeled microglial cells in the contralateral hemisphere **(I)** is similar to 1-day post-FPI. In the peri-lesion area **(J)**, the microglial response is decreased relative to the 3-day timepoint, although there is still a substantial appearance of activated microglial cells. At 30 days post-FPI **(K,L)**, the Iba1-labeled microglial cells in the contralateral hemisphere **(K)** appear to be depleted. Of the few remaining Iba1-labeled microglial cells, many continue to exhibit an activated morphology. Similarly in the peri-lesion region **(L)**, there is a noticeable lack of Iba1-labeled microglial cells in layers I–IV in the region subjacent to where the lesion occurred. In layers V and VI, there are considerably more Iba1-labeled microglial cells, some of which exhibit varying degrees of activation. Scale bar in **(B)** = 50 μm for images **(A–L)**. Scale bar = 50 μm in images **(A1–L1)**.

In order to examine the glial response following FPI in mice, we performed immunohistochemistry for astrocytes using anti-GFAP and microglial cells using anti-Iba1. The general pattern of the glia was such that at 1-day post-FPI, there were astrocytes with processes extending toward the lesion (Figure 3), indicative of astrocytes migrating to the injury site. However, in the peri-lesion area, only minimal GFAP-labeling is observed (Figure 3). Minimal GFAP-labeling was also observed throughout ipsi and contralateral cortex (Figures 4 and 5). By 3 days after FPI, there was an intense astrocyte activation located in the peri-lesion region (Figure 3) that was considerably more pronounced and widespread compared to 1 day post-FPI (Figure 3). In addition to the peri-lesion area, activated astrocytes were robustly observed throughout the entire ipsilateral cortex in the FPI mice (Figure 4), although there was only minimal GFAP-labeling in the contralateral hemisphere (Figure 4). At 7 days post-FPI, the number of GFAP-positive astrocytes was decreased throughout the ipsilateral cortex relative to the 3-day timepoint (Figures 3 and 4). In the peri-lesion area, a robust number of hypertrophied astrocytes are still observed (Figure 3), although considerably less astrocytes are seen relative to the 3-day timepoint. In the ipsilateral cortex, GFAP-labeling is observed, but not in the contralateral cortex (Figure 4). Overall, at 7 days post-FPI, there appears to be less GFAP-labeled astrocytes relative to the 3-day timepoint (Figures 3–6).

#### Iba1

**Figure 7:**
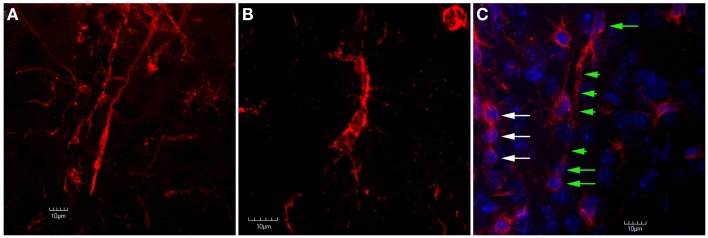
**Confocal images of trains of Iba1-labeled microglial cells in the peri-lesion area**. In **(A)**, two separate trains of microglial cells are shown. In these trains, the microglial cells exhibit a rod-like appearance. Ziebell et al. (2012) have described a similar appearance of trains of rod-like cells in the somatosensory cortex following fluid percussion injury in rats. Similar to Ziebell et al. (2012), the rod-cells observed in the present study (both single and in trains) appeared perpendicular to the pial surface. In **(B)**, despite the lack of an elongated, rod-like appearance, the cells within this train appear to have their apical and basal processes connected. In **(C)**, Iba1-labeled cells are shown in tissue that has been counterstained using DAPI. Note that there are at least three rod-cells (green arrows) within this train that contain relatively long apical and basal processes (green arrowheads). Another train of cells that does not exhibit this rod shape (white arrows) is seen at the left edge of the image. Although these cells do not exhibit a rod shape, they still appear to be connected by their apical and basal processes. Scale bars in all images = 10 μm.

At the 1-day timepoint, activated microglial cells are only sparsely observed in the peri-lesion area (Figure 6). No changes were apparent in the contralateral hemisphere. Microglial activation was most robust at 3 days post-FPI (Figure 6). The majority of activated microglial cells are observed in the peri-lesion zone (Figure 6), but to a lesser extent, were also observed throughout the ipsilateral cortex (Figure 6). At 1 and 3 days after FPI, some of the microglial cells exhibited a rod shaped morphology (Figure 7). Some of these rod shaped cells were in pairs or small trains of cells, similar to that observed by Ziebell et al. (2012). By 7 days post-FPI, activated microglial cells were only observed in the immediate peri-lesion region (Figure 6) and were relatively less abundant than the 1 and 3 day post-FPI timepoints. At this time point and beyond, very few if any of the microglial cells exhibited a rod shaped appearance (data not shown). Activated microglial cells were relatively sparse at the 30-day timepoint in both the ipsilateral and contralateral hemispheres (Figure 6).

### Second-hit PTZ Seizures

In response to the PTZ challenge, eight out of the nine mice from the FPI group exhibited stage IV/V seizures compare to one mouse in the sham group and no mice in the naïve control group (Table 1). Contingency table analysis was performed and the Chi-Square results revealed a significant increase in the development of stage IV/V seizures between the FPI group, compared to the sham group (*χ*^2^ = 10.888; *p* < 0.001), and the Naïve group (*χ*^2^ = 10.37; *p* < 0.001). There was no difference observed between sham and naïve control groups (*χ*^2^ = 0.598; *p* = 0.439, NS).

**Table 1:** **Chi-Square results from contingency table analysis of stage IV/V seizures**.

	Stage IV/V seizure	No stage IV/V seizure
FPI	8*	1
Sham control	1	8
Naïve control	0	5

Note that there is no significant differences between sham control and naïve control groups. *Denotes significant difference from sham control and naïve control group (*p* < 0.005).

The frequency of seizures was also significantly different between the three groups such that there was an increase in the FPI mice when compared with mice from the sham (*χ*^2^ = 11.455; *p* < 0.001) and naïve (*χ*^2^ = 11.455; *p* < 0.001) groups (Table 2). There was no difference observed between sham and naïve control groups (*χ*^2^ = 0.498; *p* = 0.455, NS). In addition, FPI altered the total duration of seizures between the three groups (*F* = 4.90; *p* < 0.033), such that the FPI mice had a significantly greater total duration of seizures relative to naïve (*p* < 0.049) but not relative to sham (*p* = 0.35) groups (data not shown).

**Table 2:** **Chi-square results from contingency table analysis of median seizure frequency**.

Treatment groups	Seizure frequency (median)
FPI	7*
Sham control	0
Naïve control	0

Naïve control was not different from sham control. *Denotes significant difference from sham control and naïve control group (*p* < 0.005).

## Discussion

The results from this study demonstrate increased seizure susceptibility in mice at 30 days after FPI. This finding is entirely novel and has not been reported in mice at the 30-day timepoint after TBI. It is pertinent to note that a recent study did demonstrate increased seizure susceptibility in mice after a FPI, but that study looked at the 6-month post-FPI timepoint (Bolkvadze and Pitkänen, 2012). The two studies also contain several other important differences in methodology. First, the present study used a lower subconvulsive PTZ dose of 30 mg/kg, compared with 50 mg/kg used in the aforementioned study (Bolkvadze and Pitkänen, 2012). Second, the present study delivered a more moderate trauma (1.5–1.7 atm for 12–16 ms) compared with ∼2.9 atm for 21–23 ms (Bolkvadze and Pitkänen, 2012). It should be noted that previous studies in rat have shown that increasing the pressure level of the FPI, increases the extent of tissue damage, acute impairment, and the probability of post-traumatic epilepsy (Curia et al., 2011). However, since very few studies have examined FPI in mice, it is unclear what pressure level is required to produce epileptogenic effect. The data presented in the current study show that a moderate FPI is capable of increasing seizure susceptibility, a hallmark of the epileptogenic progression. Third, the coordinates and size of the burr hole used in the two studies is different. In the present study, the burr hole diameter is 2 mm, compared to Bolkvadze and Pitkänen (2012) in which a 3-mm burr hole was drilled. Moreover, the present study used the coordinates (antero-posterior: +1.5 mm; medio-lateral: −1.2 mm), whereas the previous study did not indicate specific coordinates, rather that study indicated that it was the area over the left parietotemporal cortex between bregma and lambda. These latter coordinates are considerably more lateral compared to the current study. Moreover, a shift in the location of the craniotomy is associated with a alteration to the resulting lesion, such that more lateral coordinates are associated with an increased ipsilateral tissue damage in rats (Vink et al., 2001). Nevertheless, previous studies in the rat have shown that coordinates analogous to the ones used in the current study result in a robust pro-epileptogenic response (D’Ambrosio and Perucca, 2004; D’Ambrosio et al., 2004).

Histopathological examination of the tissue at several timepoints post-FPI revealed a stereotypical lesion, followed by scarring and a robust glial response. The glial response has clearly been initiated by 1 day post-FPI and peaks at 3 days post-FPI (Figures 2–6), as observed by astrocyte and microglial cell staining. By 7 days after FPI, a moderate level of healing has occurred such that the integrity of pial surface has been mostly restored. There are fewer activated astrocytes and microglial cells, and Fluoro-Jade labeling is considerably diminished relative to the 1- and 3-day timepoints. One cannot rule out the possibility that the decreased Fluoro-Jade labeling at 7 days post-FPI is as much a result of cells that have died, as it is cell that have been rescued. Consistent with this notion, a persistent area of necrotic tissue is evident in the region immediately surrounding the impact zone. It is pertinent to note that stereological cell counts for neurons was beyond the scope of this study, but it is entirely possible, if not likely, that neuronal loss and remodeling persists following FPI. By 30 days after the FPI, superficial examination of the tissue reveals only minor necrosis and the pial surface appears to have been repaired.

Although this study does not directly assess infiltrating components of neuroinflammation, the fact that there is blood brain barrier breakdown following FPI, as well as a robust astroglial and microglial response, is typically indicative of acute neuroinflammation (Streit et al., 2004). Moreover, we have previously performed analogous studies in the rat using the FPI paradigm and showed a robust inflammatory response in the cortex by 24 h after injury (Mukherjee et al., 2011). Previous studies examining molecular correlates of inflammation following traumatic brain injuries have indicated that the drilling of the Burr hole alone is sufficient to cause inflammation and mild levels of neuropathology (Cole et al., 2011). Therefore, the present study incorporated the use of both, a sham group that received identical treatment to the FPI group, minus the actual FPI delivery, as well as a naïve control which had no experimental manipulations performed. In the sham groups, there was no noticeable lesion at any of the timepoints examined, nor was there appreciable astrocyte or microglial activation. Despite this lack of a lesion, or glial activation, it is still possible that neuroinflammatory proteins are altered in response to the craniotomy in sham mice. The fact that one animal in the sham group did exhibit stage IV/V seizures following the PTZ second-hit challenge supports the idea that the sham surgery has the potential to not only cause an inflammatory response (Cole et al., 2011), but may also increase seizure susceptibility (Galic et al., 2012). This idea is further supported by the fact that previous studies have shown that some inflammatory proteins are pro-convulsive (Kramer et al., 2012; Vezzani and Granata, 2005). Such findings underscore the need to incorporate both, a sham and a naïve control group when performing studies that pertain to inflammation and/or seizure effects of TBI.

In conclusion, this study demonstrates increased seizure susceptibility to a sub-threshold dose of PTZ at 30 days following a FPI. Taken together with the study from Bolkvadze and Pitkänen (2012), future studies can be carried out using transgenic mouse strains and the FPI method to further elucidate mechanisms of TBI.

## Conflict of Interest Statement

The authors declare that the research was conducted in the absence of any commercial or financial relationships that could be construed as a potential conflict of interest.
